# Interaction and association analysis of malting related traits in barley

**DOI:** 10.1371/journal.pone.0283763

**Published:** 2023-04-04

**Authors:** Irfan Iqbal, Zeratsion Abera Desta, Rajiv Kumar Tripathi, Aaron Beattie, Ana Badea, Jaswinder Singh

**Affiliations:** 1 Plant Science Department, McGill University, Quebec, Canada; 2 Crop Development Centre, University of Saskatchewan, Saskatoon, Canada; 3 Brandon Research and Development Centre, Agriculture and Agri-Food Canada, Brandon, Canada; Murdoch University, AUSTRALIA

## Abstract

Barley is considered as a foundation of the brewing and malting industry. Varieties with superior malt quality traits are required for efficient brewing and distillation processes. Among these, the Diastatic Power (DP), wort-Viscosity (VIS), β-glucan content (BG), Malt Extract (ME) and Alpha-Amylase (AA) are controlled by several genes linked to numerous quantitative trait loci (QTL), identified for barley malting quality. One of the well-known QTL, QTL2, associated with barley malting trait present on chromosome 4H harbours a key gene, called as *HvTLP8* that has been identified for influencing the barley malting quality through its interaction with β-glucan in a redox-dependent manner. In this study, we examined to develop a functional molecular marker for *HvTLP8* in the selection of superior malting cultivars. We first examined the expression of *HvTLP8* and *HvTLP17* containing carbohydrate binding domains in barley malt and feed varieties. The higher expression of *HvTLP8* prompted us to further investigate its role as a marker for malting trait. By exploring the 1000 bp downstream 3’ UTR region of *HvTLP8*, we found single nucleotide polymorphism (SNP) in between Steptoe (feed variety) and Morex (malt variety), which was further validated by Cleaved Amplified Polymorphic Sequence (CAPS) marker assay. Analysis of 91 individuals from the Steptoe x Morex doubled haploid (DH) mapping population revealed CAPS polymorphism in *HvTLP8*. Highly significant (*p*<0.001) correlations among ME, AA and DP malting traits were observed. The correlation coefficient (*r*) between these traits ranged from 0.53 to 0.65. However, the polymorphism in *HvTLP8* did not correlate effectively with ME, AA, and DP. Altogether, these findings will help us to further design the experiment regarding the *HvTLP8* variation and its association with other desirable traits.

## Introduction

Barley is one of the most important cereal crops used globally as food, feed for livestock, and in the brewing industry. Barley grains are the main raw material to produce malts, which in turn are processed to produce beer in the liquor industries. Malting quality traits of barley varieties are highly desirable to produce premium quality beer. Diastatic Power (DP), Viscosity (VIS), β-glucan content (BG), Malt Extract (ME) and Alpha-Amylase (AA) are some of the key parameters for determining the malting quality of the barley [1,2]. ME is composed of all the soluble elements of malt such as carbohydrates, proteins, and their hydrolyzed products. Moreover, it is a key source of fermentable sugars and essential enzymes for the hydrolysis of starch [3]. Mixed linked (1–3, 1–4) β-glucans (hereafter “β-glucan”) constitute the major non-starch polysaccharides component of endosperm and aleurone cell walls of barley seed [4]. Among different cultivars, barley grain is the fundamental source of β-glucan content ranging between 3.4% and 5.7% [5]. β-glucan concentration in barley grains (4–10% w/w) is substantially higher compared to wheat (1% w/w) [6], which also has decent utility in the brewing industry.

Barley varieties with higher β-glucan content are desirable to the food industry as it serves as dietary fiber, which protects against various human health conditions such as lowering the blood cholesterol [7,8]. However, for the production of high-quality products through efficient malting practices, cultivars with low β-glucan concentrations are required by the brewery industries [9]. Higher levels of β-glucan can contribute towards issues like haze formation, viscous wort and reduced wort filtration during the brewing processes [10]. ME is a complex quantitative trait that is controlled by multiple genes and its concentrations can be variable in different cultivars. It is also considered as a mega-trait which is the product of interactions between many sub-trait [11,12].

Genetic manipulation and selection of malting quality traits such as ME and β-glucan content are challenging for the breeders due to the complex inheritance nature of these traits.

Quantitative trait loci (QTL) analysis has been utilized as a molecular tool to detect and estimate genomic regions that are associated with traits of interest such as malting quality traits. To date, outcomes of several studies revealed the identification of more than 250 malting quality-related QTLs. Specifically, QTLs with high variance for ME and β-glucan content have been identified and localized on all the different barley chromosomes [11]. From these QTLs, QTL2 on chromosome 4H is reported as a major barley malting quality QTL which contributes 29% and 38% variation for key malting quality parameters such as BG and ME, respectively [13]. Similarly, QTL2 is also known to have an effective influence on other malting quality traits including AA and DP [1,14]. In another study, the telomeric region of chromosome 4H containing the malting quality associated complex was fine mapped, which revealed a total of 15 putative QTLs for BG [2], ME [3], AA [6] and DP [1,4]. Another major QTL, *QMe*.*NaTx-2H* was identified by using the doubled haploid (DH) population located on chromosome 2H that accounts for 48.4% of the total phenotypic variation (R^2^) for the ME [15]. Another QTL, *Qme1*.*1* present on chromosome 1H has contributed with R^2^ of 21.1% in the ME phenotype [16]. In addition, two closely positioned QTLs were identified on chromosome 4H which accounts for the R^2^ about 8–13% and 4–10%, respectively [17]. β-glucan is also a very important malting quality trait that is influenced by both the environment and genotypic factors, but the latter has a more significant contribution, relatively [18]. Members of the *cellulose synthase-like* (*CslF*) gene family have been reported to play role in the β-glucan synthesis [19,20]. Several efforts have been made to develop molecular markers to select barley varieties with better malting traits such as ME and β-glucan content. For instance, around 1,524 SNPs were genotyped to detect several genes that are associated with six malting traits including β-glucan content and ME [21]. Further, Randomly Amplified Polymorphic DNA (RAPD), Diversity Arrays Technology (DArt) and QTL-related PCR-based makers have been used to screen different barley populations for improved malting quality traits [2,22–25].

Recently, QTL2, a locus with a large proportion of variation for ME and β-glucan content, was dissected which harbours a key gene *HvTLP8*, apparently involved in interacting with β-glucan in a redox-dependent manner [26]. In the present study, we made efforts to characterize *HvTLP8* at the molecular level to develop functional markers that can help barley breeders to identify varieties with superior malting traits. These markers include variation at DNA and protein levels. Moreover, we examined the linear association between the marker and the barley malting traits (e.g., ME, AA, and DP) as well as among these traits.

## Materials and methods

### Plant material and growth conditions

The seeds of barley malt and feed varieties (Table 1) were received from Drs. Aaron Beattie (University of Saskatchewan), Ana Badea (Agriculture and Agri-Food Canada Brandon Research and Development Centre), and Plant Gene Resources of Canada (PGRC).

**Table 1:** List of barley malt and feed varieties.

Sr. No.	Name	Row-type	Purpose
1	Champion	Two	Feed
2	CDC Maverick	Two	Feed
3	CDC Bow	Two	Malt
4	CDC Meredith	Two	Malt
5	CDC Austenson	Two	Feed
6	CDC Fraser	Two	Malt
7	AC Metcalfe	Two	Malt
8	CDC Copeland	Two	Malt
9	CDC Cowboy	Two	Feed
10	CDC McGwire	Two	Feed
11	TR12735	Two	Feed
12	CDC Kindersley	Two	Malt
13	Polar Star	Two	Malt
14	AAC Synergy	Two	Malt
15	Steptoe	Six	Feed
16	Morex	Six	Malt

Seeds were grown in the greenhouse under the photoperiod regime of 16 hrs day and 8 hrs night with an average temperature of 20°C. Fresh leaf samples were collected for DNA extraction and stored in -80°C for future use. Mature seeds of two malting (AC Metcalfe and Morex) and two feed (Steptoe and CDC Cowboy) were surface sterilized with a 20% working concentration of bleach followed by three rinses with distilled water. Seeds were germinated at room temperature under dark on wet Whatman filter paper in sterile petri-plates. Samples were collected and flash-frozen in liquid nitrogen at 16 hrs of grain germination stage. The harvested samples were stored at -80°C for future experiments.

### Total RNA isolation and DNase I treatment

Total RNA was isolated from the 16 hrs germinated grains using a Spectrum Plant Total RNA kit, following the manufacturer’s protocol (Sigma-Aldrich, St. Louis, MO, USA). Before cDNA synthesis, extracted RNA samples were subjected to DNase I treatment to avoid DNA contamination by using the RQ1 RNase-Free DNase kit (Promega, USA). Reaction mixtures were incubated at 37°C for 30 minutes (mins) followed by the addition of 1μl of RQ1 DNase stop solution for reaction termination. Reactions were further incubated at 65°C for 10 mins to inactivate DNase I.

### cDNA synthesis and quantitative real-time PCR (qRT-PCR) analysis

First-strand cDNA from 1μg of DNase I treated total RNA samples was synthesized by following the recommended protocol of AffinityScript QPCR cDNA Synthesis Kit (Agilent Technologies, USA). Relative transcript levels were measured by qRT-PCR using Wisent advanced qPCR master mix (Wisent Bioproducts, Canada) on Mx30005p qPCR system (Stratagene, USA). The qPCR cycle conditions were 95°C for 2 mins; 40 cycles of 95°C for 5 seconds (sec) and 60°C for 30 secs. For each sample, two independent biological and three technical replicates were used. Relative transcript levels were analyzed by following the 2^-ΔΔCq^ method [27] using *HvActin* as an internal reference control [28]. The significance of gene expression between different malt and feed varieties was measured by all pairs Tukey test at (P ≤ 0.05). Integrated DNA Technologies (IDT) primer quest tool (https://www.idtdna.com/PrimerQuest/Home/Index) was used to design the primers for *HvTLPs* (*HvTLP8* and *HvTLP17*). The primer sequences are listed in (Table 2).

**Table 2:** Primers used for qRT-PCR expression analysis.

Primer names	Primer sequences
*HvTLP8_qFP*	CACATTGCCCAATTGTAGATAGC
*HvTLP8_qRP*	AGCTCCTAAACTAGCGGTG
*HvTLP17_qFP*	GACAATTGGCAGCATTCATC
*HvTLP17_qRP*	GATCCTTTGGGCATGTACTC
*HvActin_qFP*	GCCGTGCTTTCCCTCTATG
*HvActin_qRP*	GCTTCTCCTTGATGTCCCTTA
*HvGAPDH_qFP*	GTGAGGCTGGTGCTGATTA
*HvGAPDH_qRP*	CGTGGTGCAGCTAGCATTTGAGAC

### DNA extraction, PCR amplification and gel electrophoresis

Leaf samples (50 mg) were frozen in liquid nitrogen and grounded by using tissue lyser (Qiagen, USA). DNA extraction was performed by following the modified phenol/chloroform method as described by Singh et al. [29]. Primers (*HvTLP8_F*: ATGCCATTCTTCCTCACCACAG and *HvTLP8_R*: TCATGGGCAGAAGATGAC) were used to amplify the coding sequence of *HvTLP8*. Primers (*HvTLP8_3’UTR_F*: CGAGCACACGGACAAGAATA and *HvTLP8_ 3’UTR_R*: GCAACGACTCCAGTGAACTTA) targeting the 1000 bp downstream of *3’ UTR* region of *HvTLP8* were used. PCR amplification was performed in a 20 μl reaction, containing 1 μl of gDNA for each sample. PCR amplification was performed using GoTaq^®^ G2 green master mix (Promega, USA). The PCR conditions were 95°C for 2 min, followed by 36 cycles at 95°C for 30 seconds with the annealing temperature of 56°C. A 20 μl of amplified product was analyzed on 1.2% agarose gel.

### Cloning and CAPS assay

Amplified fragments of *TLP8* were extracted from agarose gel and purified by using the recommended protocol of the Nucleospin gel and PCR clean-up kit (Takara, USA). Purified TLP8 fragments were ligated into the pGEMT-easy vector by using the manufacturer’s protocol. The ligation mixture was transformed into the DH5α competent cells. Several colonies were inoculated for plasmid isolation. Plasmid extraction was performed by using the modified TIANs method. The quality of plasmid was determined by using the NanoDrop ND-1000 (NanoDrop Technologies, Wilmington, DE, USA). Next, the clones were confirmed by PCR and restriction digestion. The positive clones were further sent for Sanger sequencing to Genome Quebec (https://cesgq.com). Sequence alignments were performed by using the Multiple Sequence Comparison by Log-Expectation (MUSCLE; ebi.ac.uk/Tools/msa/muscle/) tool with the default settings. For CAPS assay, the amplified fragments of all samples were digested with the MwoI enzyme (NEB, USA). Digested products were electrophorized on 2% agarose gel to visualize the polymorphism.

### Malting traits data and *HvTLP8* variation

A doubled haploid (DH) mapping population (Table 3) derived from a cross between Steptoe (S) x Morex (M) [30] was used to investigate the polymorphism in *HvTLP8*. These two parents (Steptoe and Morex) were grown in controlled environment as described above. The young leaves were collected for DNA extraction. The protocols for DNA extraction, PCR amplification, CAPS assay was performed as described in the previous method sections above. Morex (malting variety) and Steptoe (feed variety) are two contrasting parents used for the traits under investigation. Morex has higher ME, AA and DP as compared to Steptoe. Data of DHs based on means for three different malting traits (ME, AA, and DP) over nine environments was retrieved from the GrainGenes database (https://wheat.pw.usda.gov/ggpages/SxM/phenotypes.html). Correlation analysis between *TLP8* marker and malting quality traits as well as other traits described above was performed as described in [31].

**Table 3:** Information about the S x M lines and *HvTLP8* allele variation in the S x M DH mapping population.

Sr. No.	SxM no.	*HvTLP8* allele	Sr. No.	SxM no.	*HvTLP8* allele	Sr. No.	SxM no.	*HvTLP8* allele
1	SM1	A	32	SM40	B	63	SM77	A
2	SM2	A	33	SM41	B	64	SM78	A
3	SM3	A	34	SM42	B	65	SM79	B
4	SM4	B	35	SM43	A	66	SM80	B
5	SM5	B	36	SM44	B	67	SM81	A
6	SM6	A	37	SM45	B	68	SM82	B
7	SM7	A	38	SM46	B	69	SM83	A
8	SM8	A	39	SM48	A	70	SM84	A
9	SM9	A	40	SM50	B	71	SM85	B
10	SM10	A	41	SM54	A	72	SM87	A
11	SM11	B	42	SM55	A	73	SM88	A
12	SM12	A	43	SM56	A	74	SM89	A
13	SM13	B	44	SM57	B	75	SM91	B
14	SM14	B	45	SM58	A	76	SM92	B
15	SM15	B	46	SM59	B	77	SM93	A
16	SM16	B	47	SM60	B	78	SM94	B
17	SM17	A	48	SM61	B	79	SM98	A
18	SM20	A	49	SM62	A	80	SM99	A
19	SM21	B	50	SM63	B	81	SM110	B
20	SM22	A	51	SM64	A	82	SM114	A
21	SM23	A	52	SM65	B	83	SM116	B
22	SM24	A	53	SM67	A	84	SM120	B
23	SM26	B	54	SM68	B	85	SM124	A
24	SM27	A	55	SM69	B	86	SM125	A
25	SM29	B	56	SM70	B	87	SM126	A
26	SM30	A	57	SM71	B	88	SM127	B
27	SM31	A	58	SM72	A	89	SM129	B
28	SM32	B	59	SM73	A	90	SM130	B
29	SM35	B	60	SM74	A	91	SM131	A
30	SM38	A	61	SM75	A	92	Steptoe (Parent)	Allele A (Steptoe)
31	SM39	A	62	SM76	B	93	Morex (Parent)	Allele B (Morex)

## Results

### Differential expression of *HvTLPs* in malt and feed varieties

**Fig 1:**
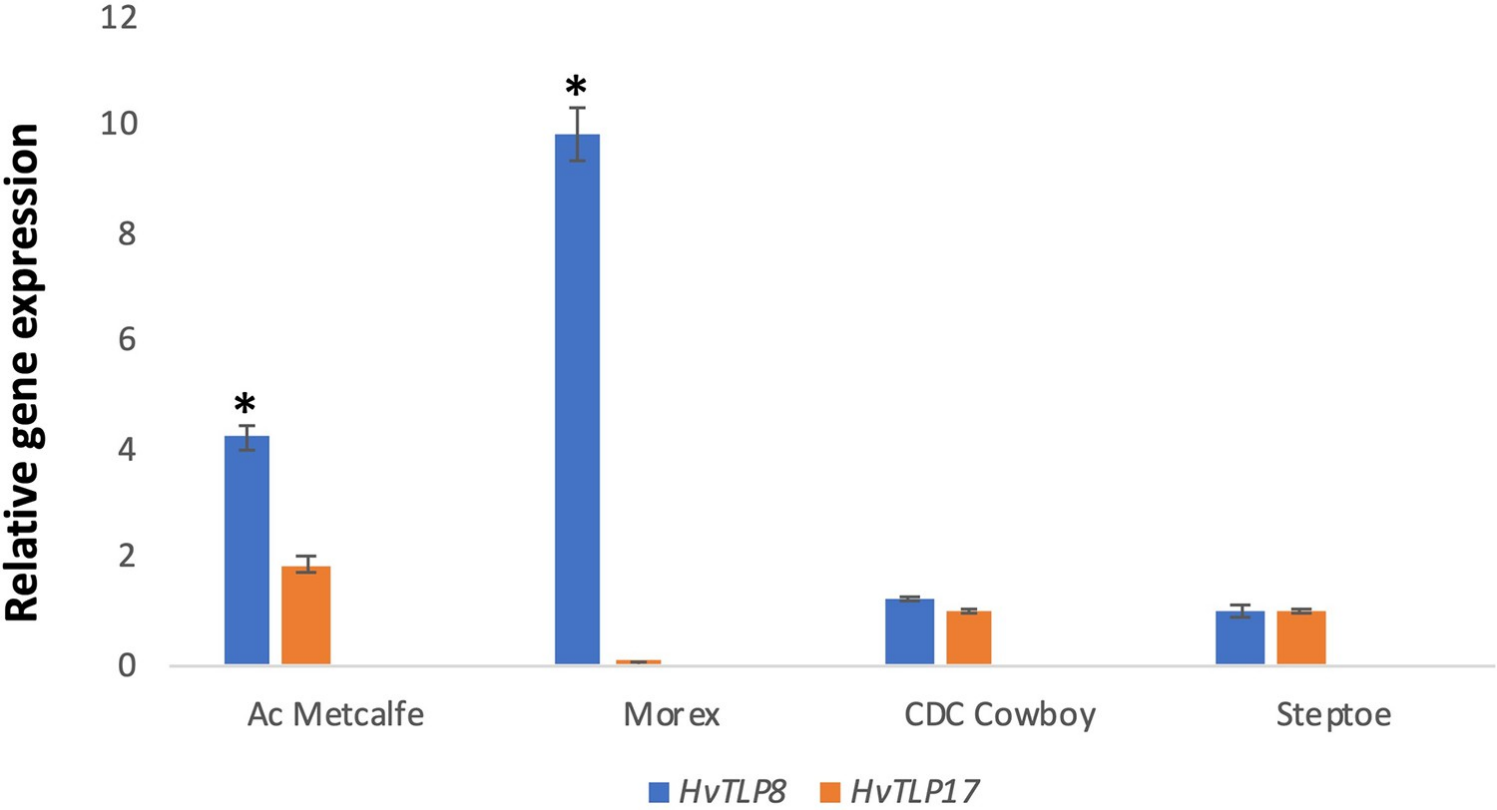
Expression analysis of *HvTLP8* and *HvTLP17* in malt (AC Metcalfe and Morex) and feed (CDC Cowboy and Steptoe) varieties at 16 hrs of grain germination. Asterisks (*) represent the significance of gene expression in different malt and feed varieties by measured by all pairs Tukey test at (P ≤ 0.05).

Our previous genome-wide analysis of the barley genome identified 19 *TLPs* some of which possesses carbohydrate binding domain (CBD) [32]. The role of carbohydrate binding domain in TLP8 has been documented for its interaction with beta-glucan [26]. Therefore, we selected CBD containing TLPs, *HvTLP17* and *HvTLP8* to examine the expression during germination. The expression of *HvTLP8* was higher in malting (AC Metcalfe & Morex) varieties as compared to feed varieties. Among malting varieties, the highest *HvTLP8* expression was observed for Morex. However, lower and similar expression was observed for both of the feed varieties Steptoe and CDC Cowboy. In the case of *HvTLP17*, we have observed higher expression in only one malt (AC Metcalfe) variety and lower expression in feed varieties at the 16 hrs stage of grain germination (Fig 1).

### Variation in the coding sequence and in 3’ UTR region of *HvTLP8*

**Fig 2:**
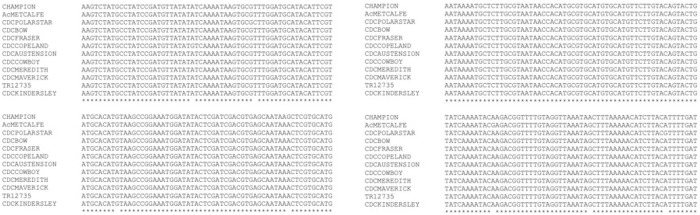
Multiple sequence alignment of 1000 bp downstream 3’ UTR region of *HvTLP8* from different malt and feed varieties.

**Fig 3:**
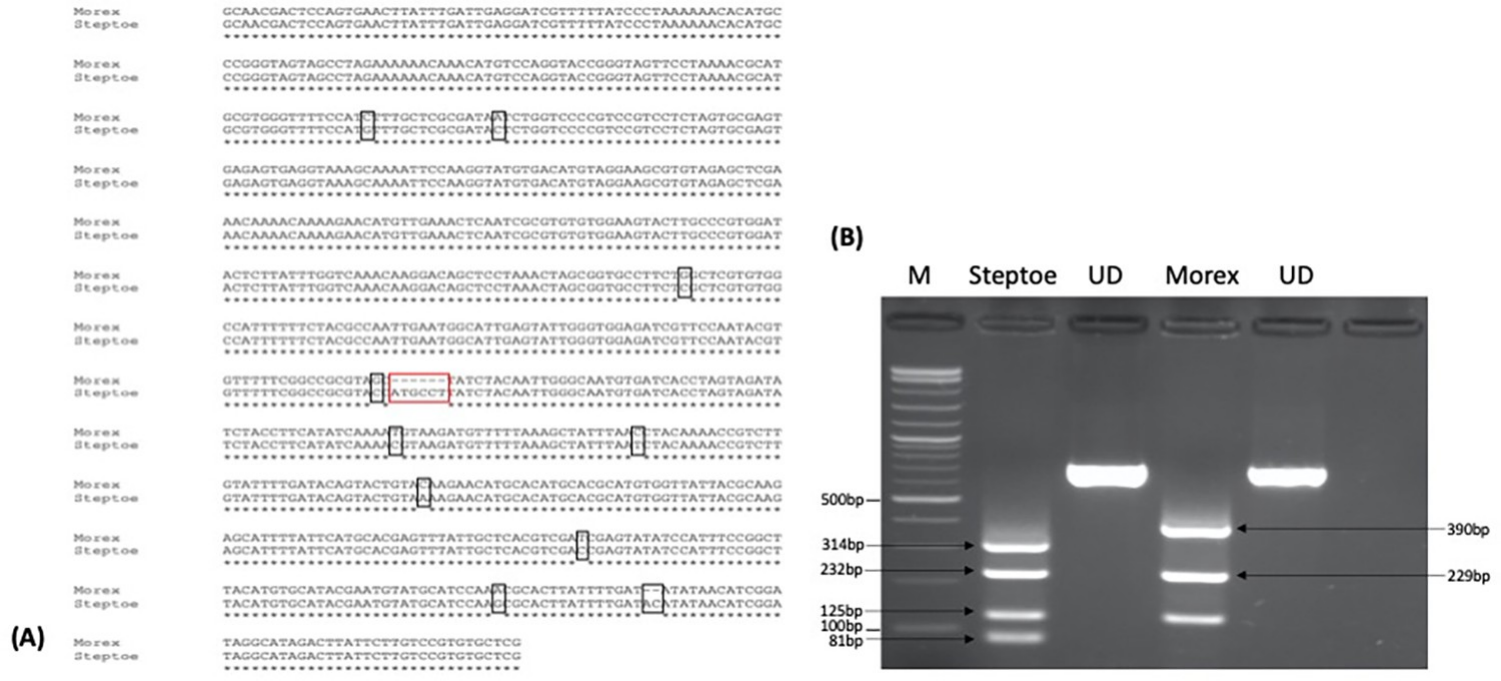
**(A)** Alignment of 1000bp downstream 3’ UTR region of *HvTLP8* in Steptoe (six-row feed) and Morex (six-row malt) varieties. The red box indicates the difference that resulted in the additional MwoI restriction site in Steptoe. Black boxes indicate SNPs. **(B)** Digestion of 1000 bp downstream 3’ UTR region of *HvTLP8* fragment with MwoI in Steptoe and Morex. UD: Undigested PCR product. M: 1kb plus marker.

**Fig 4:**
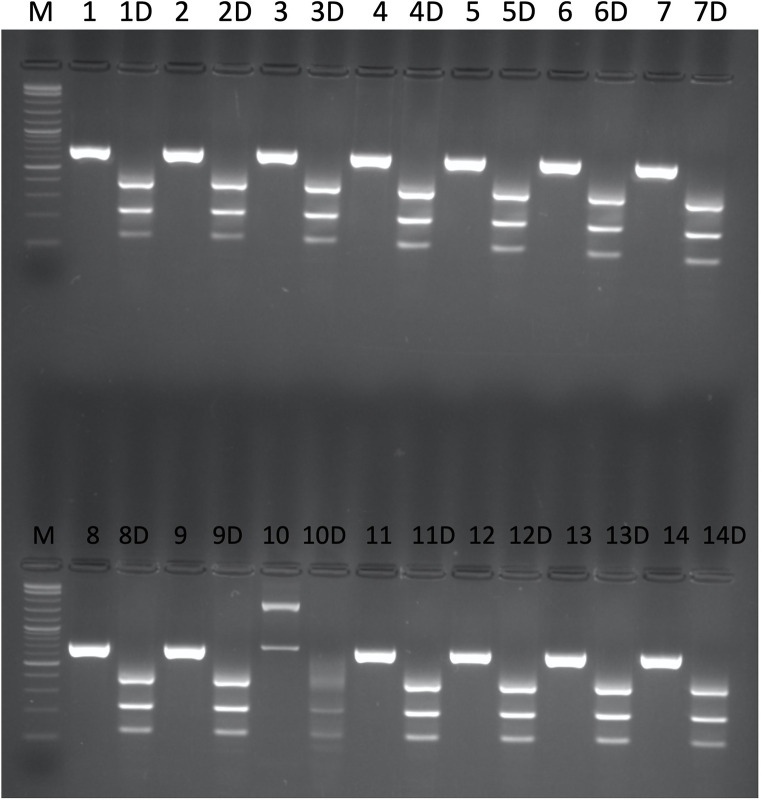
Digestion of 1000 bp downstream 3’ UTR region of *HvTLP8* fragment with MwoI in different barley malt and feed varieties (1: Champion, 2: CDC Maverick, 3: CDC Bow, 4: CDC Meredith, 5: CDC Austenson, 6: CDC Fraser, 7: AC Metcalfe, 8: CDC Copeland, 9: CDC Cowboy, 10: CDC McGwire, 11: TR12735, 12: CDC Kindersley, 13: CDC Polarstar, 14: AAC Synergy). D: Digested PCR product. M: 1kb plus marker.

Based on *HvTLP8/17* expression data we decided to explore *HvTLP8* as the marker for malting quality. First, we PCR amplified the coding regions of *HvTLP8* from malt and feed varieties and sequenced. Alignment of the sequenced amplified fragments from different malt and feed varieties indicated no polymorphism for the *HvTLP8* coding region (data not shown). Next, we decided to sequence the un-translated regions (UTRs) of *HvTLP8*. We searched for polymorphism to the 1000 bp downstream of the 3’ UTR region and were unable to identify any conserved variation specific to malting or feed varieties in this region (Fig 2). However, the sequencing results indicated differences in the sequence of *HvTLP8* 3’ UTR region in between Steptoe (six-row feed) and Morex (six-row malt), which was striking. Polymorphism included eight single bp SNPs, a two bp deletion in Morex and a six bp difference in Steptoe, which resulted in additional site development of MwoI restriction enzyme (Fig 3). When amplified fragments of the *HvTLP8-* 3’ -UTR region was subjected to digestion with MwoI electrophoresed samples indicated a clear discriminative banding pattern in Steptoe and Morex varieties (Fig 4). Digestion of Steptoe PCR amplified *Hv*TLP8 fragments generated four bands of 81 bp, 125 bp, 232 bp and 314 bp sizes, whereas Morex *Hv*TLP8 fragments produced three bands of 125 bp, 229 bp and 390 bp sizes (Fig 3).

### Association of SNP variation present in 3’ UTR of *HvTLP8* with malting quality traits

**Fig 5:**
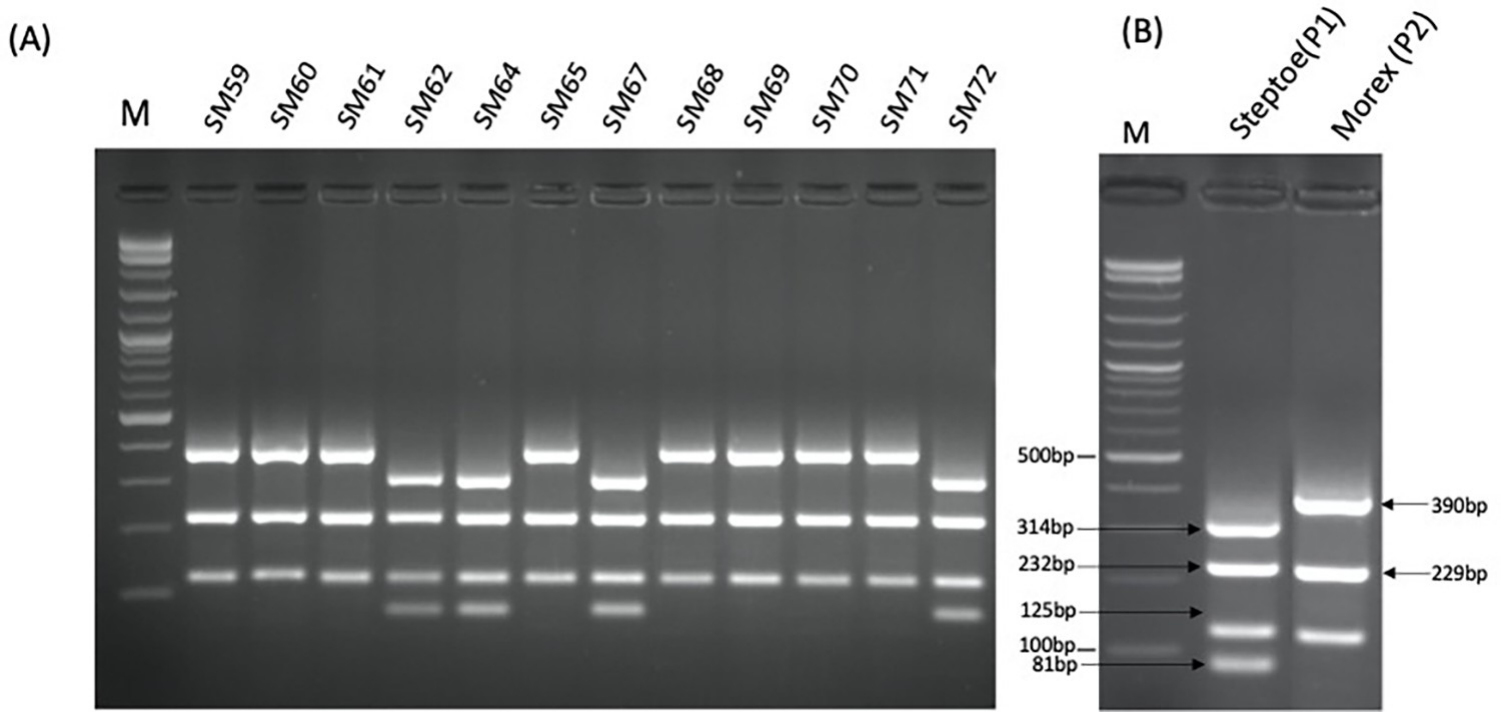
**(A)** Digestion of 1000 bp downstream 3’ UTR region of *HvTLP8* fragment with MwoI in DHs from S x M mapping population. **(B)** Digestion of mapping population parents. M: 1 kb plus marker.

Our discovery of SNP variation present between the 3’ UTR of *HvTLP8* of Steptoe and Morex (Fig 3A), prompted us to elucidate the genetic variation of these SNPs in different mapping populations. We used a DH mapping population generated from the cross between S x M genotypes to analyze the SNP variation for *HvTLP8*. By performing the CAPS assay on 91 DHs and the parents, we found polymorphism for *HvTLP8* among lines (Fig 5A and 5B). Individuals mimicked 52.75% of the allele A genotype of Steptoe (P1), whereas 47.25% of individuals demonstrated the allele B genotype, which corresponded to Morex (P2) (Table 3).

**Fig 6:**
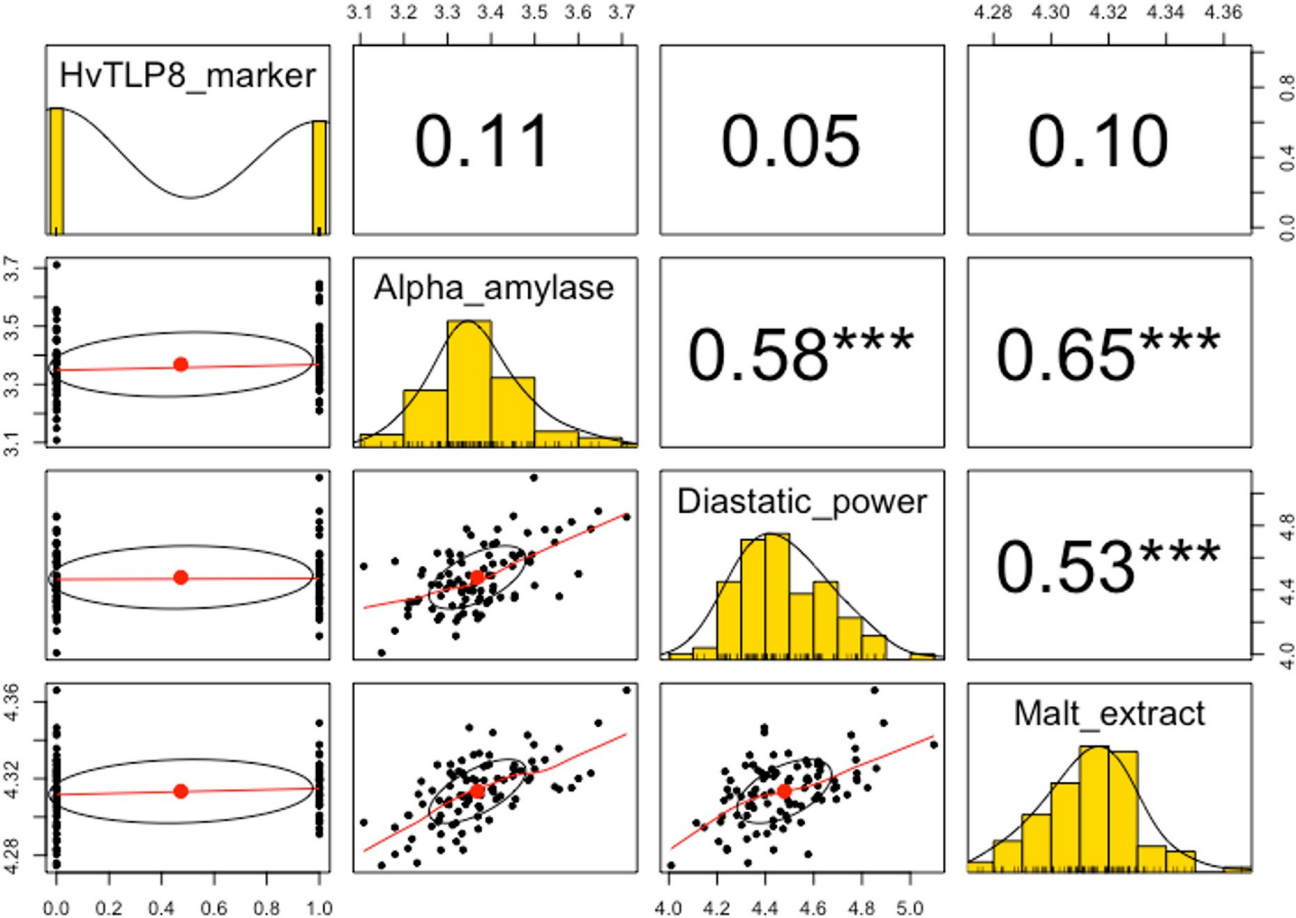
Correlations (*r*) between *HvTLP8* and malting quality traits along with the correlation between traits for DH mapping population. The scatter plot matrix (below diagonal), the histogram distributions are in the diagonal, and the correlation values are above diagonal. The (*) represents labels of significances (***) shows highly significant correlation (*p*<0.001) and values without asterisks are insignificantly correlated.

S x M DH mapping population is well studied for malting traits especially for ME, AA and DP [12]. *HvTLP8* demonstrated polymorphism in the S x M mapping population, which intrigued us to examine its relationship with ME, AA, and DP. Regression analysis indicates that *HvTLP8* did not explain the variation for these traits. The observed R^2^ values were 1.32, 0.94 and 0.29 for AA, ME, and DP respectively (Table 4). Similarly, correlation analysis revealed that *HvTLP8* was insignificantly associated with these traits; however, the correlation among AA, ME and DP were highly significant (*p*<0.001; Fig 6). The highest correlation was found between AA and ME (0.65). The correlation coefficient (*r*) between traits ranged from 0.53 to 0.65 (Fig 6).

**Table 4:** The proportion of variation (%) shared between the malting traits of S x M mapping population.

Malting Traits	R^2^ (%)	P-value
Alpha amylase	1.32	0.273
Malt extract	0.94	0.355
Diastatic Power	0.29	0.603

## Discussion

The molecular basis of barley malting quality trait plasticity is poorly understood. Here we study the behaviour of *TLP8* as a marker for barley malting quality. In our earlier report, *HvTLP8* was identified as a key gene that influences the malting quality *via* interaction with β-glucan in a redox-dependent manner [32]. We observed that TLP8 contained the carbohydrate-binding motif and its expression was differential in different barley malting and feed varieties at both mRNA and protein levels, respectively [26]. Our other recent finding reported 19 *HvTLPs* in the barley genome other than *HvTLP8* and only two germination specific TLPs, HvTLP8, and HvTLP17 contained the carbohydrate-binding motif (CQTGDCQG) and can have a possible interaction with the β-glucan [33]. In addition to these two TLPs, HvTLP14 also possesses partial carbohydrate binding motif [32] as glycine (G) was substituted to glutamine (Q), therefore removed from further investigations. Thus, we examined the mRNA expression of *HvTLP8* and *HvTLP17* (Fig 1). The expression analysis data indicated that *HvTLP17* has a higher gene expression level in the malting variety, AC Metcalfe, than in the Morex. However, the expression of *HvTLP*8 was higher in malt than in feed varieties. The expression pattern of *HvTLP17* was different from *HvTLP8* in this study and also in the earlier report [26]. However, HvTLP17 has a carbohydrate binding motif similar to HvTLP8 [33]. It is possible that HvTLP17 might be binding with other carbohydrates but not with beta-glucan. These results indicated that *HvTLP8* might be a good candidate gene to develop a potential molecular marker that can differentiate between malt and feed barley varieties. To investigate this, we analysed the full-length coding regions (CDS) of *HvTLP8* to find potential SNPs that can be associated with malting. We could not observe SNPs between the CDS region of *HvTLP8* in different malt and feed varieties. Nevertheless, we found differences at gene expression level for *HvTLP8* in different malt and feed varieties, when the regions from the CDS and 3’ UTR were targeted [26]. Next, we expanded our SNP exploration targeting the 1000 bp downstream of *HvTLP8* 3’ UTR regions in different malt and feed varieties. Interestingly, we observed SNP polymorphism in two six-row varieties Steptoe and Morex (Figs 2, 3A and 3B). We observed several polymorphic SNPs in *HvTLP8* of Steptoe and Morex. However, a six bp deletion in the 3’UTR region of *HvTLP8* in Morex was one of the major polymorphic sites identified. Previous investigation on SNP discovery in the reference genome of barley by using 16,127 assemblies have reported higher SNP density in the 5’UTR region compared to the 3’ UTR region [34]. In another study, SNP polymorphism analysis of the *HvP5CS1* gene revealed 16 SNPs, from which 7 SNPs were found in the 3’ downstream sequence of the non-coding region [35]. Likewise, our results of SNP discovery in the *HvTLP8* 3’ UTR also exist in the downstream region as found in these reports.

Varieties having higher ME, DP, AA, and lower BG traits are considered malting varieties which are an absolute requirement for the malting industry. The malting quality of barley depends on the interaction of various malting traits and the genetic architecture of genotypes. To fish out the genes for malting, numerous malting quality associated QTLs have been identified in barley. Among these QTLs, QTL2 accounts for 37.6% of the variation for the malt extract [13]. The *HvTLP8*, resides on the QTL2 present on chromosome 4H of the barley genome. Steptoe and Morex are two contrasting six-row barley varieties for malting traits such as ME, DP, AA, and BG. The polymorphism observed in the 3’UTR of *HvTLP8* was further tested on S x M DH mapping population that is well studied for the malting traits [1,12,30]. We performed CAPS marker assay on 91 DHs and found polymorphism for *HvTLP8* in 1000 bp downstream of the 3’ UTR region (Figs 4, 5A and 5B). A total of 52.75% of DHs showed a closer relationship with Steptoe, while 47.25% were related to Morex (Table 3). Furthermore, our regression analysis on malt quality parameters demonstrated low variance of *HvTLP8* with changing AA, DP, and ME (Table 4). Similarly, correlation data indicated a low association of *HvTLP8* with these malting traits. However, a significant correlation was observed between AA, DP, and ME (Fig 6). QTL2 is a complex genetic region harbouring genes for different malting traits, which are known to interact with each other [30] and *HvTLP8* may only be associated with soluble beta-glucan content, which has not been considered in this study. Polymorphism identified in the *HvTLP8* could be correlated with other traits such as beta-glucan content mapped in the QTL2 region to develop potential genetic markers. Our data analysis showed very low deviation for the AA and DP values, as it was reported by Ulrich et al. [12]. However, the correlation values between traits were found to be higher (Fig 6) when compared to the results (r = 0.56** and 0.39**) reported by Ulrich et al. [12] referring to the correlations between AA and ME as well as between DP and ME, respectively. This could be due to the availability of analysis tools having better algorithms and the way of analysis which have been used (*p*<0.001) for comparison (*p*<0.01) as reported by Ulrich et al. [12].

Our current data and previous studies [32,33] suggested that the coding sequence of *HvTLP8* is completely similar in different malt and feed varieties. However, expression of this gene varies greatly between malt and feed varieties. Difference in the gene expression needs further investigation to identify regulatory elements in the promoter region, which may provide further information about its transcriptional regulation. We also noticed that purified HvTLP8 was interacting with carbohydrate moiety at the protein level [26], therefore any marker technology which differentiate gene and protein expression could be utilized for selection of better malting quality barley genotypes. For example, markers, that deploy proteins and enzymes (e.g., ELISA-based) could provide better options. ELISA-based markers have been used for the screening of traits like lysine content in wheat [36], levels of deoxynivalenol (DON) [37] and Hordein (Gluten) [38] in beer samples. In future, development of HvTLP8 specific antibodies to develop an ELISA based biochemical marker will help the barley malting breeding community for selection of superior barley malting varieties.

## Conclusion

In this study, we have explored the CDS and 3’ UTR (1000 bp downstream region) of *HvTLP8* for SNP variation in different malting and feed varieties that can be linked to malting. We found SNP variation in the 3’ UTR downstream region of *HvTLP8* of Steptoe and Morex. Importantly, we found a six bp deletion in the Morex variety as a key variation. Similarly, we found polymorphism while exploring the 3’ UTR of *HvTLP8* (1000 bp downstream) in the DH population. Our correlation analysis indicated that *HvTLP8* was insignificantly correlated with malting traits (ME, AA, and DP), however, highly significant correlations were recorded for these traits at (*p*<0.001). The identified SNPs in the *HvTLP8* can be further characterized to reveal their possible association with the malting traits. In future, the 5’ UTR upstream region could be explored for the potential *HvTLP8* variation that may have a possible association with key malting traits.
